# Decisional Needs of People From Minority Ethnic Groups Around Living Donor Kidney Transplantation: A UK Healthcare Professionals’ Perspective

**DOI:** 10.3389/ti.2023.11357

**Published:** 2023-07-24

**Authors:** Ahmed Ahmed, Anna Winterbottom, Shenaz Ahmed, John Stoves, Sunil Daga

**Affiliations:** ^1^ Department of Renal Medicine, Leeds Teaching Hospitals NHS Trust, Leeds, United Kingdom; ^2^ Leeds Institute of Health Sciences, Faculty of Medicine and Health, University of Leeds, Leeds, United Kingdom; ^3^ Division of Psychological and Social Medicine, Leeds Institute of Health Sciences, University of Leeds, Leeds, United Kingdom; ^4^ Department of Renal Medicine, Bradford Teaching Hospitals NHS Foundation Trust, Bradford, United Kingdom; ^5^ Leeds Institute of Medical Research, Faculty of Medicine and Health, University of Leeds, Leeds, United Kingdom

**Keywords:** transplantation, living donor, advanced kidney disease, ethnic minorities, decision making

## Abstract

Despite improved patient and clinical outcomes, living donor kidney transplantation is underutilized in the United Kingdom, particularly among minority ethnic groups, compared to deceased donor kidney transplantation. This may in part be due to the way in which kidney services present information about treatment options. With a focus on ethnicity, semi structured interviews captured the views of 19 kidney healthcare professionals from two renal centres in West Yorkshire, about the decisional needs and context within which people with advanced kidney disease make transplant decisions. Data were analysed using thematic analysis. Themes were categorized into three groups: 1) Kidney healthcare professionals: language, cultural awareness, trusted personnel, and staff diversity, 2) Patient information resources: timing and setting of education and suitability of patient-facing information and, 3) People with advanced kidney disease: knowledge, risk perception, and cultural/religious beliefs. To our knowledge, this is the first study in the United Kingdom to investigate in depth, healthcare professionals’ views on living donor kidney transplantation decision making. Six recommendations for service improvement/delivery to support decision making around living donor kidney transplantation among minority ethnic groups are described.

## Graphical Abstract

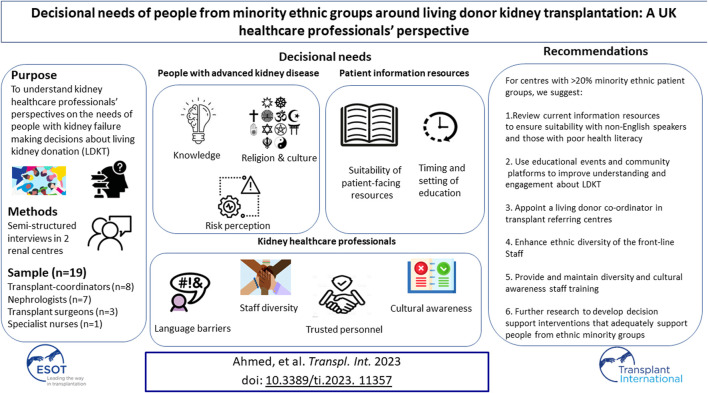

## Introduction

Living donor kidney transplantation (LDKT) has superior patient and clinical outcomes for people with advanced kidney disease (AKD), including better quality of life, survival, graft success compared to deceased donor transplantation, and is more cost effective than dialysis [1, 2]. Despite this, few people with kidney disease receive a live donor transplant compared to other renal replacement options; [3], and ethnic and socio-demographic differences in the uptake of LDKT are reported; [4]. AKD is up to five times more common among minority ethnic groups due to a higher prevalence of long term conditions such as diabetes mellitus and hypertension [5, 6]. In the United Kingdom (UK), 27% of people on renal replacement therapy (RRT) are from minority ethnic groups [6]. More than half of transplant centres in the UK have >20% of their waiting list from people from ethnic minority groups with a third of these centres with >20% people from South Asian heritage [6, 9]. Yet the ethnic diversity of living kidney donors in United Kingdom (UK) has remained the same between 2006 and 2017 [7]. South Asians, the second largest ethnic group in the UK [8], receive only 17% of live donor kidney transplants compared to 33% for White and 11% for Black ethnic groups [9]. The disproportionately low number of organ donors from these groups results in longer waiting times for a deceased donor, and worse outcomes because of longer periods of dependence on dialysis treatments [10, 11].

National frameworks recommend timely preparation of people with AKD for renal replacement therapy (RRT), options including LDKT. This includes offering balanced, accurate information about all forms of RRT and how they may impact on people’s lives [12]. However, these guidelines do not address how variations in practice might impact on treatment uptake rates (by ethnicity), nor do they identify which interventions are most effective in helping to prepare people to make treatment decisions [13–15]. Several challenges exist for kidney services providing decision support as outlined below:

1) People making decisions about LDKT are presented with multiple treatment decisions often considered simultaneously, i.e., dialysis modality decisions, alongside decisions about deceased donor and living kidney donor transplantation. Each treatment has multiple different options, attributes, and consequences [16, 17]. Some patient information and patient decision aids present these treatment options equally, despite LDKT having optimal patient and clinical outcomes and the potential to forgo the need of dialysis with pre-emptive transplantation [18]. It is unknown how transplantation options should be described in patient information to accurately reflect how services present these to individuals with AKD [13]. Significant systemic changes and new ways of thinking are required to increase the uptake LDKT and furthermore to achieve it prior to the need for dialysis treatments [19].
2) Patient leaflets are most used to support face to face discussions within consultations. Quality assessments of this information suggests that it is presented in a way that is difficult to understand, does not signpost to cultural/religious relevant information and focusses more on preparation for surgery and treatment and/or service information that is not relevant to decision making [20–22].
3) People with AKD seeking LDKT take an active role in seeking and approaching potential donors. To do so, they must have knowledge about the transplantation process. This may be particularly challenging for people from ethnic minorities, as health literacy rates, i.e., people’s ability to read, understand and act upon health information, are often low [23, 24]. This may in part explain why some people prefer to adopt a “watch and wait” approach in the hope of being called up for a deceased kidney donor transplantation [25, 26]. Designing interventions to support people with low health literacy may improve people’s understanding and decision making [27].

To date, decision support interventions for people making LDKT decisions have been developed in non-UK settings and address various aspects of the decision making process, including interventions targeting recipient [28–30] or donor education [31, 32], decision coaching [33], and decision aids for healthcare providers [34]. In the UK, whilst various groups have started to explore decisional needs for LDKT, these studies have lacked diversity in terms of ethnicity and inclusion of non-English language speakers [35, 36]. Our team are undertaking research studies to develop an understanding of the decision needs for LDKT decision making in a diverse population including non-English speakers and minority ethnic groups, particularly South Asians [37]. This exploratory research aims to understand the decisional needs of people from minority ethnic groups in relation to LDKT from the perspective of kidney healthcare professionals (HCPs). This will increase our understanding about the type(s) of interventions that can enhance LDKT decision making.

## Methods and Materials

### Design

This study employs a qualitative methodology using semi-structured interviews with kidney HCPs. Research governance approvals via the Health Research Authority and NHS Research Ethics committee were granted in June 2020 (Reference: 21/NW/0095).

### Setting

The study was conducted at Leeds and Bradford renal units in West Yorkshire, UK. The Leeds Renal Unit is the regional transplanting centre and oversees the care of 1,200 kidney transplant recipients and 450 living donors, with around 200 transplants performed annually. The Bradford Renal Unit is a transplant referral centre and provides care for 430 kidney transplant recipients and approximately 50 living donors. There are 150 patients active on the national transplant waiting list for Leeds and Bradford centres, combined. Around 40% and 18% of people with AKD on RRT in Bradford and Leeds respectively are from minority ethnic groups [3].

### Sample

Non-probability sampling was employed [38]. Participants were eligible to take part if they met the following inclusion criteria: Kidney HCPs directly involved in assisting people with AKD in making LDKT decisions. The following groups were not eligible to participate: Kidney HCP with no direct involvement in transplantation, those who support living-donors or paediatric patients and colleagues (authors) directly involved in conduct of this research.

### Recruitment

**FIGURE 1:**
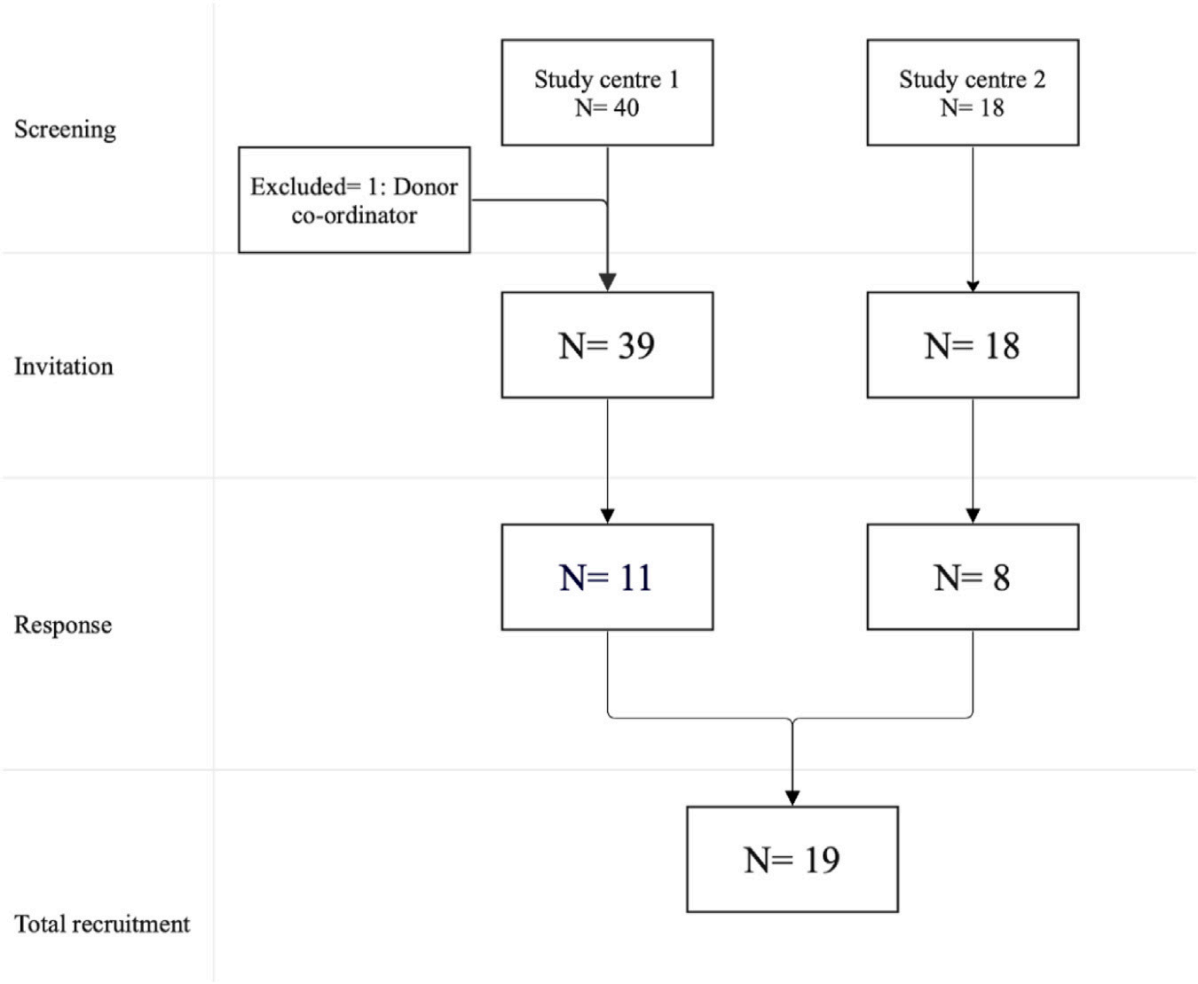
Consort diagram of recruitment process of participants.

Eligible participants were contacted via NHS email. Figure 1 describes the recruitment process in the study centres.

### Study Materials

An interview guide was developed using published literature on people decision making about LDKT and guided by the expertise of the research team and relevant stakeholders from each renal centre. The interview guide contained three parts: 1) Introduction: Briefing, demographics and describing clinical context 2) Exploration of views: HCP perspectives on patient’s decisional needs and patient information resources supporting LDKT decision making 3) close: opportunity to add any additional comments. For further information see Supplementary Material.

### Data Collection

Semi-structured interviews were conducted virtually via Microsoft Teams (version 4.2.4.0) by researcher (AA) and took an average of 41 min (range 27–63 min). Participants were given the opportunity to ask questions about the study and provided their written consent to take part, before the interview. Data were transcribed using Otter software (Otter.ai, Indigo, 2.2.22/26 June 2020). Interviews were conducted until saturation of themes [39].

### Data Analysis

Interviews were analysed using thematic analysis [40]. NVivo software (QSR International, release 1.7.1) was used to manage the data.

## Results

The characteristics of the participants (P) are described in Table 1.

**TABLE 1:** Sample participant characteristics.

Participants’ characteristics	Transplanting centre N = 11	Referral centre N = 8
Sex		
Female (F)	6	5
Male (M)	5	3
Ethnicity		
White	10	4
South Asian	1	4
Clinical Role		
Nephrologist	4	3
Transplant surgeon	3	0
Transplant co-ordinator	4	4
Specialist nurses	0	1

Themes are categorised under three broad headings: a) Kidney HCPs, b) patient information resources c) people with AKD.

### Kidney HCPs

#### Language Barriers

HCPs believed that communication with non-English speaking patients could be difficult.

“We have language deficits; I think that's a massive issue. Clearly, we have a major second language probably Urdu /Hindi variant” P *3, F, Transplant co-ordinator*

In these instances, hospital interpreters and family members were used to support discussions, and HCPs preferred hospital interpreters because of their familiarity with medical terminology. However, hospital interpreters were also felt to be time consuming, and less likely to provide accurate and complete translations.

“I think it's difficult to get information across, however good the translator is, and when some time is spent in translation, it means that maybe you haven't got the time to focus on those other important things, like live donation” *P 11, M, Transplant surgeon*

#### Cultural Awareness, Trusted Personnel and Staff Diversity

HCP highlighted several ways in which services could improve the delivery of information to people from ethnic minority groups who are making LDKT decisions. These included, improving the ethnic diversity of front-line staff, trusted personnel to improve communication, engagement with the kidney services and overcoming cultural barriers in LDKT decision making.

“I'd be very honest to say that all the transplant coordinators are from white ethnicity. Having someone from same community who has a real understanding of the issues that affect donation in that community, would be useful” *P 6, M, Consultant nephrologist*

“Let's put the effort in, before we approach patients, to get them to engage we need to know how living donation sits within their culture” *P, 14, Female, Specialist nurse*

A lack of trust was thought to relate to peoples’ prior experiences with health services, as well as more generalised concerns about how people of different ethnic groups are treated by the NHS. Furthermore, participants highlighted the need for training about gender-related cultural issues, and different religious viewpoints. One participant cited the importance of their cultural improvement officer for enhancing rapport and patient engagement.

“There may be some sort of distrust if they've kind of not grown up necessarily in this country. And I think it's very important when we meet with those patients to address some of those issues, or find out why people aren't coming forward” *P 1, F, Transplant co-ordinator*

“I think there are worries, you know, certainly at the moment, in terms of how people with different ethnicities that are treated in different areas of the NHS, you must know about the high maternity deaths among Black ethnic groups” *P 4, F, Transplant co-ordinator*

### Patient Information Resources

#### Timing of Education

HCPs suggested that information and education designed to prepare people for making decisions about renal replacement therapies should be provided at an earlier stage of the kidney disease pathway. They recognised that people with AKD are often asked to consider different treatment options, e.g., dialysis and transplantation at the same time, and that it would be beneficial to have more time to consider the options.

“When they are in low clearance clinic, they are already symptomatic, you are asking them what option do you want? dialysis? what sort of dialysis? Ok let me tell you about transplant. But you need to make a decision about dialysis as well. You need access. Do you have a live donor? too much and need to be spaced” *P 2, F, Consultant nephrologist*

#### Setting of Education

HCPs saw value in providing group education sessions to help people make treatment decisions and dispel misconceptions about transplantation, rather than in one-to-one consultations with nephrologists and transplant co-ordinators. They proposed a variety of settings such as the renal unit, primary care and community and faith-based community settings.

“I have attended community events where we talked about and promoted transplantation and donation, people feel more comfortable sometimes [to go] somewhere [where] they are used to [going] and [engaging] in different discussions” *P 1, F, Transplant co-ordinator*

#### Suitability of Patient Facing Information

HCP use information leaflets to supplement their discussions with patients. They valued these resources’ ability to provide people with AKD with basic treatment information. However, they believed that this generic information might not be appropriate for non-English speakers and those with lower levels of health literacy. Apart from one booklet in Urdu, HCPs reported that the current written resources are all in English. It was suggested that resources should be translated into regional languages, particularly for people from South Asia, as this ethnicity makes up a significant proportion of their local population.

“I think in somewhere like [Study centre 2] where there's a large Asian population, it will be a great help for this literature and booklets to be available in the local language, predominantly local language” *P 15, M, Consultant nephrologist*

Additionally, HCPs had concerns about the readability of the patient facing materials they use to supplement their LDKT discussions.

“Potentially, some patients may find them difficult to understand, their language sometimes can be a little bit complex for some people, they need to be simplified to suit more people” *P 19, F, transplant co-ordinator*

HCPs felt that patient-facing materials should include clarification of common misconceptions about the transplantation process, and information related to different cultures and religions in relation to transplantation.

“We are all concerned about live donation in South Asians and Muslims, but those books don’t really [talk] about live donation in Islam. Or how helping someone else sits with that culture” *P 14, F, Specialist nurse*

“Sometimes little things can make big difference, for example displaying a cartoon picture of a Sikh person with a turban can make people more trusting and willing to know more about treatments like this” *P, 14, Female, Specialist nurse.*

Furthermore, HCPs thought that it was valuable for people with AKD to talk to other people in a similar situation to share their experiences

“Sometimes people find it easier if they see the story of someone who had the same journey” *P 4, F, Transplant co-ordinator*

### People With Advanced Kidney Disease

#### Knowledge About LDKT

HCPs believed that people with AKD do not have enough knowledge about the advantages and disadvantages of LDKT, alternative treatment options, the transplantation process, donor-work up and donation suitability, to make an informed decision. They believed that knowledge gaps are more prevalent among people with low levels of health literacy particularly those with low education, low socioeconomic status and the non-English speakers.

“You know, we have many of the non-English speaking patients around this region, also some have low education and bad social circumstances. They are low in literacy, they may not have the required knowledge, they even sometimes have wrong knowledge about live donation” *P 3, F, Transplant co-ordinator*

#### Risk Perception

Healthcare professionals felt that this lack of knowledge about transplantation could lead to concerns about the short- and long-term consequences of transplantation, the physical risk of an operation and the financial implications of donation.

“Because of what they have been through with kidney disease, some people have genuine concerns about how someone could have a healthy life with one kidney, those are the ones who won’t ask their family even if that means they stay on dialysis forever if they don’t get a kidney from the list” *P 7, M, Consultant nephrologist*

“They do often worry and ask how long till their donors are able to work? What about their job, etc. And specially when it's a donor coming from abroad, they worry about airfare and loss of earnings” *P 12, M, Consultant nephrologist*

#### Cultural and Religious Beliefs

Healthcare professionals identified instances where they felt that cultural and religious beliefs impacted on decision making. For example, Muslims were thought to require greater clarification about their religion stance on donation.

“Islam in the Great Britain is not a homogenous entity. So, communities are very dependent on what their own Imam thinks. Certainly, some Imams don't take the same lead as the Muslim Council of Great Britain. So, I suppose there are a lot of different perceptions about living donation” *P 6, M, Consultant nephrologist*

Healthcare professionals also suggested that in their experience, people from ethnic minorities were less likely to trust the health service, and people from South Asia were more private and less willing to discuss their health and had concerns about others perception of a woman’s suitability for marriage after donation.

“So sometimes we have kind of media campaigns, Our South Asian patients will not consider this, obviously, not a lot of people like that, but they like to keep more private” *P 4, F, Transplant co-ordinator*

“It tends to be older women from the Asian community who end up donating, they worry if a young girl donated a kidney, she's somehow seen as less suitable when it comes to marriage” *P 15, M, Consultant nephrologist*

## Discussion

This study identifies themes that HCPs believed were important in supporting people to make decisions about LDKT. Some of the themes pertain to the individual characteristics of people with AKD, such as knowledge, religion, and culture, whereas others, such as the way in which education about LDKT is delivered (including timing and setting), are linked to the range and availability of resources that may assist them in making transplant decisions. While some themes are thought to be shared by all ethnic groups, others such as knowledge gaps were thought to be more prevalent among non-English speakers and those with lower socioeconomic status. The multitude of these attributes within minority ethnic groups add another layer of complexity when considering tailored interventions to improve LDKT uptake [60]. Understanding how to support people with AKD who need to make treatment decisions requires an appreciation of the different goals, values, knowledge, skills and motivation of the key stakeholders who support the decision making process, including families/carers and HCPs [41, 42]. There are few qualitative studies assessing the perspective of HCPs each with a difference focus, including African American populations patient level [43], communication barriers [44], and interventions to improve access to LDKT using existing models [45] (Table 2). To our knowledge, we have conducted the first UK based study that explores the views of HCPs about the decisional needs of minority ethnic groups around LDKT, with a view to developing a culturally sensitive decision support intervention.

**TABLE 2:** Summary of qualitative studies exploring HCPs’ perspective and LDKT.

Study	Shilling et al. [43]	Sandal et al. [44]	Bailey et al. [45]
Country	USA	Canada	UK
HCPs number	18	16	15
Type	Focus group	Semi-structured interview	Semi-structured interview
Focus	Patient level barriers	Barriers in discussing LDKT with patient	Development of multicomponent intervention from existing interventions
Overlapping themes	Medical mistrust Knowledge Risk perception	Language, Cultural barriers	Cultural barriers and resource limitations
Recommendation	Further research on tailored educational program	Policy changes to inform health delivery systems of targeted and effective intervention	Evaluate the multicomponent intervention in RCT

Consistent with other studies, we found that religious and cultural beliefs and trust are consistently reported as major barriers to people pursuing LDKT, particularly in minority ethnic groups [46, 47, 61]. Information should signpost to religious and cultural information relevant to transplantation that is available in patient-facing resources [20] and within community outreach and informal promotions. The latter associates with a higher number of people pursuing LDKT [44]. In the UK, community- and faith-based platforms have been used to address cultural and religious barriers to LDKT [48]. These outreach interventions have increased awareness and interest in LDKT however there has been only a limited effect on uptake rates [49].

Furthermore, our study participants highlighted the need for diversity training to improve cultural and religious awareness of factors that might impact on people’s willingness to pursue transplantation. Similar conclusions were made in a Dutch study that examined HCPs engagement with culturally diverse populations [61]. Providing regular training should improve skills and confidence over time, rather than reinforce stereotypes and leave staff feeling overcautious and uncertain and in their ability to communicate with people from ethnic minorities [50, 51]. A regular programme of staff training also is important to maintain quality of education [62]. Moreover, as recommended in the National Health Service (NHS) people plan [53], employing ethnically diverse front-line staff who are more representative of the local population can support people with AKD by improving engagement and trust in medical services [52].

Written patient information is commonly used by HCPs to supplement discussions and support shared decision-making [20]. There is considerable variation in its quality [20, 21] and HCPs in our study expressed the view that this information is not suitable for non-English speakers and people with low health literacy, who are often from minority ethnic groups [21]. Signposting people to resources produced in alternative and multi-lingual formats, use of interpreters or bilingual staff may address these language and health literacy needs [54, 61]. Such tailored resources (culturally sensitive written information and videos) have only recently been developed by community organisations working with patient groups and HCPs, including the sharing of experiences of other people with AKD who have previously made a decision about LDKT into educational sessions. The need to implement the use of these resources was advocated by participants in this study. This has already been recognised nationally with inclusion of resources in the NHS Blood and Transplant “Transplant TV” series [55], the National Black, Asian and minority ethnic (BAME) Transplant Alliance initiative [63], and other projects that support LDKT decision making [56, 57]. Recent guidance on the inclusion of narratives in patient decision aids interventions, suggest that whilst they may be beneficial to help people understand others experiences of the process with which a decision was made, they may also bias people’s decision making [58].

The timing and setting of patient education about LDKT is another important aspect of decision making. There is a wide variation in practice between renal units within the UK, with many large non-transplanting centres undertaking the work-up and evaluation of potential living kidney donors locally rather than referring them to the transplanting centre. Delays at various stages of the living donor pathway contribute to lower LDKT rates in non-transplanting renal centres, and the presence of Living Donor Coordinators (LDCs) in these centres would facilitate more timely decision making. National Health Service Blood and Transplant (NHSBT) and the UK Living Kidney Donor Network have developed a LDC workforce calculator to support the commissioning of LDCs [63] but implementation remains a challenge. In addition, a NHSBT-led UK transplant workforce survey of staff in transplanting and non-transplanting UK centres is in progress and this may help to identify unwarranted variation in staffing resource between centres.

This study recruited a range of kidney health professionals working within two large inner city kidney units; however, the findings may be limited in their generalisability to other UK kidney units as the diversity of these units may represent a proportion of the population with different needs to that as a whole. It is promising however that a national workshop involving multi-centre HCPs at the 2022 UK Living Kidney Donor Network meeting identified the same top three resource-related barriers to LDKT (cultural issues, language, and health literacy) [59].

Based on the study findings we recommend the following measures to improve LDKT decision making for people from diverse ethnic groups in units where minority ethnic groups represent >20% of the deceased donor waiting list:

1. Review current patient information resources to ensure their suitability for people particularly with low health literacy and non-English speakers, including signposting to culturally tailored information involving those communities that are most disadvantaged [20, 55, 63].
2. Explore ways in which the experience of other people with AKD can be used in educational events and platforms [55–57] to improve understanding and health literacy without biasing people’s decision making [58].
3. Appoint living donor co-ordinators in transplant referral centres as per national guidance and supported by the NHSBT LDC workforce calculator [63]. This will facilitate a dedicated and proactive LDC role within all renal centres and therefore reduce unwarranted variation in practice.
4. Enhance the ethnic diversity of the frontline staff such as transplant coordinators by affirmative recruitment, for better engagement of ethnic minority groups with kidney services and to improve the quality of decision support.
5. Develop and maintain a regular programme of diversity and cultural awareness staff training that addresses all of the issues pertinent to transplantation and organ donation.
6. Further research and review of the current evidence base to develop tailored decision support interventions that adequately support people from ethnic minority groups.

## Data Availability

The datasets presented in this article are not readily available because informed consent included data collection solely for this study purpose. Requests to access the datasets should be directed to AA, ahmed.ahmed30@nhs.net.
