# Supplementary material for: Decisional Needs of People From Minority Ethnic Groups Around Living Donor Kidney Transplantation: A UK Healthcare Professionals’ Perspective
Source: Transpl Int. 2023 Jul 24;36:11357. doi: 10.3389/ti.2023.11357 (PMC10405286; doi:10.3389/ti.2023.11357)
Supplement: Supplementary file 1 [file Table1.DOCX]

| Interview part  **Healthcare professionals’ perspective on facilitators and barriers to patients’ decision making about LDKT- Interview schedule** | Briefing | Questions | Prompts |
| --- | --- | --- | --- |
| Part one:  **Introductory**  **Briefing**  **Demographics**  **Establishing clinic context** | As per preparatory document | - Can you describe your role/involvement with kidney patients when they make decisions about transplant option? - How long they have been doing this role for? - I would like to hear a bit more about how those discussions please, at what point in patients care those discussions take place? - Where? how long? and how frequent? - Who else within the team do you work closely with to help patients make decisions about transplant options? | - Do you have direct involvement in discussing transplant options with patients? - Who records the decision? is it revested? |
| Part two – Exploratory-  A  **Enquire about current resources** | Thank you, in the next set of questions we will talk about how the current resources support patients and staff in the decision making about Transplant options and LDKT | - What resources (if any) do you use to support patient’s decision making about transplant options along with consultations? - What language are they written in? Are they easy to Understand? - How do you provide written information for people who don’t speak English? - How do you feel about the support those resources provide to help people from different ethnic groups with making decisions about transplant options? - What do you think is important to discuss when talking to patients about transplant options and LDKT? - With the current resources, do you feel well prepared to cover the important aspects of that discussion with patients from different ethnicities Eg South Asians? | - Any written materials/leaflets? Websites? - Are you supported are they by interpreter services? How well does this work? - Are there any issues in relying on an interpreter or member of the family to translate? - How do you describe LDKT to the patients as an option of treatment? - What do you think is missing or could be improved in the resources? |
| B  **Decisional needs**  Part 3  Close | Thank you, the next set of questions will explore pts decision making about LDKT | - From your observations and discussions with patients, what do you think are the factors that influence their decision making? - What else do you think makes people decide to choose or not choose LDKT? - Do people usually volunteer to mention any concerns? What needs prompting? - How good do you think are the current available resources in supporting you to help patients with those concerns? - Have you come across any religious reservations about LDKT? - What about culture and what people perceive as cultural norms? Do you think this could affect decision making about LDKT? How. - Can you think of any sort of support or training needs that you think would help you cover those concerns?   Thank you for taking the time talk to me today, I have now come to the end of my questions. I there anything else you wanted to share ? | - Any specific concerns? - Are those needs different in certain groups of pts? How? - What about South Asians? - How to best prepare staff to support those conversations? Can you identify any training needs? |
